# Assessment of Physician Well-being, Part One: Burnout and Other Negative States

**DOI:** 10.5811/westjem.2019.1.39665

**Published:** 2019-02-28

**Authors:** Michelle D. Lall, Theodore J. Gaeta, Arlene S. Chung, Erin Dehon, William Malcolm, Adam Ross, David P. Way, Lori Weichenthal, Nadine T. Himelfarb

**Affiliations:** * Emory University School of Medicine, Department of Emergency Medicine, Atlanta, Georgia; † New York-Presbyterian Brooklyn Methodist Hospital, Department of Emergency Medicine, New York, New York; ‡ Maimonides Medical Center, Department of Emergency Medicine, Brooklyn, New York; § University of Mississippi Medical Center, Department of Emergency Medicine, Jackson, Mississippi; ¶ University of Louisville School of Medicine, Department of Emergency Medicine, Louisville, Kentucky; || The Ohio State University College of Medicine, Department of Emergency Medicine, Columbus, Ohio; # University of San Francisco-Fresno, Department of Emergency Medicine, Fresno, California; ** Alpert Medical School of Brown University, Department of Emergency Medicine, Providence, Rhode Island

## Abstract

Physician well-being is a complex and multifactorial issue. A large number of tools have been developed in an attempt to measure the nature, severity, and impact of both burnout and well-being in a range of clinical populations. This two-article series provides a review of relevant tools and offers guidance to clinical mentors and researchers in choosing the appropriate instrument to suit their needs, whether assessing mentees or testing interventions in the research setting. Part One begins with a discussion of burnout and focuses on assessment tools to measure burnout and other negative states. Part Two of the series examines the assessment of well-being, coping skills, and other positive states.

## INTRODUCTION

The word “burnout” was originally used by Herbert Freudenberger in 1974 to describe a state of emotional fatigue that was becoming more prevalent during the free clinic movement, attributed to the mismatch of resources to the needs of patients.^1^ Thought to be a reaction to chronic emotional and interpersonal workplace stressors, it is a three-dimensional syndrome consisting of emotional exhaustion, depersonalization or cynicism, and a sense of reduced personal accomplishment.^2^ Exhaustion from increased workloads and extended work hours combined with the stress of cognitive decision-making in the setting of emotionally-charged situations contribute to physician burnout.^3^ These causes of physician burnout have, in recent years, been only exacerbated by increased clerical workload from electronic health records and reduced sense of work efficacy.^4^

The first national study of burnout in United States (U.S.) physicians was conducted in 2011 across all specialty disciplines. From a sample of over 7,000 physicians, approximately 46% reported at least one symptom of burnout and only 49% reported satisfaction with their work-life balance. Variability was noted across medical specialties, with the highest rates of burnout noted among physicians at the front lines of access to care, such as primary care and emergency medicine (EM). This study further compared U.S. physicians to working adults in non-medical matched cohorts and concluded that physicians comparatively had both more symptoms of burnout and more job dissatisfaction than their non-physician peers.^5^

In 2014, rates of physician burnout and job dissatisfaction were compared with the results from 2011 and both were discovered to be on the rise, with 55% of physicians having reported one symptom of burnout and only 41% reporting satisfaction with work-life balance.^6^ The U.S. adult working population had not seen the same increased rates of burnout and dissatisfaction in the same amount of time, thus further increasing this disparity between physicians and non-physician working adults. This pattern of burnout has not only been identified in attending physicians, but also in resident physicians and medical students.^7^,^8^

The prevalence of burnout in the physician population is significant when taken in consideration with the effects that it has upon physicians as individuals, the patient population that physicians serve, and the institution of medicine itself. Physicians who suffer from burnout have higher rates of substance abuse, personal relationship problems, anxiety, and depression.^9^–^11^ These same physicians are more likely to self-report performing suboptimal patient care practices, such as admitting or discharging patients early, not offering options or answering questions, ordering more tests, not treating patients’ pain, not communicating important handoffs, and not discussing plans with staff.^12^ Burnout has additionally been identified as a risk factor for higher rates of medical errors, patient safety errors, and mortality ratios among hospitalized patients.^3^,^13^,^14^ At the institutional level, physician burnout has been linked with reduction in clinical care hours, which threatens to intensify the projected shortage of physicians in the year 2025.^15^,^16^

As burnout is studied more, other variables such as depression, anxiety, and stress have been identified beyond emotional exhaustion, adding to the complexity of this syndrome. There is significant overlap in symptoms between burnout and depression and anxiety. Physicians reporting burnout are at greater risk for depression and anxiety. While there is an association, suffering from burnout does not equate to a clinical diagnosis of depression.^17^ It is important to note that depression and anxiety remain mental disorders well-defined in the *Diagnostic and Statistical Manual of Mental Disorders*, fifth edition, (DSM-V) published by the American Psychiatric Association (APA) whereas burnout remains a work-related, non-DSM defined syndrome. Given that burnout is defined as a condition resulting from severe stress relative to one’s own emotional and cognitive reserves, stress has been determined to be a considerable variable in burnout assessment. Stress arising from uncertainty, risk of poor outcomes, and high-stakes environments in medical practice often leads to anxiety, which in one study of emergency physicians (EP) was the greatest predictor of career burnout.^18^

### Summary

Physician burnout is an increasingly prevalent crisis in our healthcare system, has become a focus of multiple medical organizations, and has been highlighted in both the popular media and the medical literature. The highest rates of burnout among physicians are among those specializing in primary care and EM. In the EM literature, research has shown that faculty have a poor ability to accurately identify burnout in trainees and that additional education is needed on methodology of trainee assessment.^19^ Accurate measurement is key to conducting needs assessments, developing appropriate interventions to problems, and ongoing monitoring.^20^

Numerous assessment tools are available to the EP. The goal of the Assessment Tools Workgroup, a sub-committee of the Council of Emergency Medicine Residency Directors (CORD) Resilience Committee, was to research and summarize the various assessment tools available for burnout and related factors and compile them as a collated resource. This is the first resource available in this series and will focus on assessment tools to measure burnout and other negative states. For assessment tools related to well-being, resilience, and positive states, please refer to “Assessment of Physician Well-being Part Two: Beyond Burnout.”

## METHODS

The instruments included in this article are the result of a scoping review of English-language publications with abstracts indexed in PubMed, Web of Science, and MedEd Portal within the past 10 years. Searches were based on the main Medical Library Subject Heading (MeSH) terms “burnout,” “anxiety,” and “depression.”

In addition to the search on the main term, subheadings included the following: each in quotes measurement, assessment, evaluation, diagnosis, education, etiology, trends, derivation, validation, tool, instrument, scale, measure, survey, or questionnaire and resident, residency, intern, internship, medical student, clerk, attending, physician, and clinician. A complete listing of search terms can be found in Appendix 1. This search was augmented by reviewing article reference lists and performing further citation searches. We did not include instruments cited only in abstracts or as reports of meetings.

Abstractors performed a comprehensive review of the identified assessment tools. Details of all scales and where they can be found are presented in Table and Appendix 2. The tools identified as most relevant, accessible, and practical in evaluating EP well-being were included for further review. The tools were selected by multiple abstractors. Abstractors worked in groups of two or three focused on one subject (e.g., burnout or depression). Discrepancies between abstractors were reviewed by either the first, second, or senior author. Consensus between at least two reviewers was required for an instrument to be included here.

Our primary inclusion criteria was use of the tool in a physician population in the medical literature. Exclusion criteria included tools that either were not used in a physician population or were not cited in the medical literature relating to physicians more than 2–3 times. Excluded tools that did not meet these two criteria are referenced in Appendix 2. The figure illustrates the search algorithm and tool-selection process. The articles reviewed were organized by the subcategory of the tool (e.g., burnout tools), then by individual tool, and finally, by the populations for which the tool had been used.

A summary of the scale’s purpose, structure, and evidence of its psychometric properties were derived from the original source references. Due to the varied psychometric properties of each tool, abstractors relied on the reported validity and reliability from the source manuscripts. Where available, published cutoff scores are provided for guidance, although their validity or utility in other clinical or research contexts should not be assumed. Where psychometric properties were not explicitly described in the primary sources, potential users may need to check for any subsequent information pertaining to reliability and validity.

The order of presentation is based on the following two subsections: Burnout Tools; and Depression and Anxiety Tools. The following comments and discussions should be read in conjunction with the details reported in Table and Appendix 2, as well as with the recommendations provided at the end of the review.

## RESULTS

### Burnout Tools

In the mid-1970s a group of researchers led by Christina Maslach began to seriously consider the complex and often difficult relationship that people in helping professions have with their work and the subsequent impact on their health and social networks. These researchers conceptualized burnout as a psychological syndrome in response to chronic interpersonal stressors on the job defined in three key dimensions: overwhelming exhaustion; feelings of cynicism and detachment from the job; and sense of ineffectiveness and lack of accomplishment.^21^

#### Maslach Burnout Inventory

The Maslach Burnout Inventory (MBI) is a self-assessment tool to measure experienced burnout in individuals. Across a wide range of demographics and occupations, the tool has demonstrated reliability, convergent validity, and discriminant validity.^2^,^22^ The original and most widely used version of the MBI is known as the MBI-Human Services Survey (MBI-HSS). The MBI-HSS scores participants on three distinct but inter-related subscales: emotional exhaustion; depersonalization; and diminished personal accomplishment. While the authors of the MBI consider burnout as existing on a continuum rather than as a dichotomous state, they provide population norms for some groups as a benchmark for comparing scores. The MBI suggests burnout for professionals scoring in the high range on emotional exhaustion, in the high range for depersonalization, and in the low range for personal accomplishment. Official score reports also contain information on reducing burnout and resources for seeking help.^2^

Studies on physician burnout have almost exclusively used the MBI-HSS as a measurement tool due to the large body of literature supporting its reliability.^2^,^22^ This includes many of the often-cited, population-based studies,^5^,^33^,^34^ as well as studies in residents^23^–^25^ and EPs.^25^,^26^ Consequently, the MBI-HSS has in many ways become the preferred assessment tool for a wide variety of uses, such as evaluating the effectiveness of wellness programs, faculty and resident surveillance, and demographic trends.

While the MBI-HSS has a number of strengths, there are also some limitations to its use. The high cost of administration may limit access. Users of the MBI must also consider that burnout has clear discriminant validity.^2^,^22^ In other words, it is truly a distinct phenomenon from other established constructs, such as depression and job dissatisfaction, and should not be used as a comprehensive catchall for determining individual or population mental health. It is important to note that the MBI was not normed on physicians-in-training. Attending physicians were sampled for the normative data. Finally, the MBI does not consider non-professional confounders of burnout, such as child care demands, the schedule and support of partners, life events, or financial concerns.^27^

Well-being is a complicated and multidimensional construct, and the simple absence of burnout as determined by the MBI does not necessarily equate overall well-being. Nevertheless, the MBI-HSS remains one of the most recognized, widely used, well-validated, and reputable tools in the toolbox for assessing occupational burnout in physicians and residents.

#### Single-Item Emotional Exhaustion and Depersonalization Scale

Because the MBI is a 22-item instrument, its use may be constrained by the time required for completion. To address this limitation, Shanafelt and colleagues created the single-item emotional exhaustion (EE) and depersonalization (DP) scale.^28^ These authors used the following two questions from the MBI: “I feel burned out from my work,” and “I have become more callous towards people since I took this job.” Multiple studies have significantly correlated these two questions with the EE and DP subscales of the MBI, respectively.

The single-item EE and DP scale has been well-validated in physicians, medical students, and residents with very large sample sizes.^29^ The scale is brief and free to use. However, some authors have raised concerns regarding the reliability of single-item surveys.^30^ In general, however, models that used the single-item EE and DP scale did show consistency with those who used the full 22-item MBI.^29^ This scale may be most useful for larger surveys in which only a few questions can be dedicated to burnout.

#### Copenhagen Burnout Inventory

The creators of the Copenhagen Burnout Inventory (CBI) felt the MBI could only apply to those who do “people work,” and thus could not be extrapolated to those who do not explicitly work with clients. They reported that the personal burnout section of the CBI could be applied to anyone, regardless of whether they worked with people or were employed at all.^31^ During their pilot in Danes, respondents reacted negatively toward phraseology of the MBI questions, some of which did not translate well into Danish, and the CBI creators thus sought to create a different tool altogether. The CBI was originally used in the human services sector with multiple professions, only one of which was hospital physicians. There have since been studies completed using the CBI in anesthesiologists and critical care physicians, as well as EPs. These have yielded consistent, valid, and reliable results measuring burnout.^32^–^34^

The personal burnout section asks how tired or exhausted the individual is on both physical and emotional levels, as well as how often an individual feels weak and susceptible to illness. The work-related burnout category asks to what degree an individual’s physical and psychological fatigue and exhaustion are related to his or her work. The authors stressed that they were not looking at causality, but merely how much the respondent attributed his or her stress/burnout to work. Comparison of work-related burnout and personal burnout then allows causal assessment of an individual’s burnout, be it due to work or other non-work factors such as family or health issues. The client-related burnout section determines if the respondent’s burnout is due to people-oriented work focus.^31^

The CBI, unlike the MBI, is open access and free to use. Additionally, it performs very well in assessing burnout, and also has the added benefit of looking at burnout in different aspects of an individual’s life. It has been translated into multiple languages and has been used in the physician population.^35^ Like other tools for assessing burnout, it should not be used to measure depression or an individual’s overall well-being as these are different and complex phenomena. Overall though, the CBI is a helpful tool for evaluating burnout and one that could easily be substituted for the MBI with the added benefit of being free to use.

#### Utrecht Work Engagement Scale

The Utrecht Work Engagement Scale (UWES) is derived from positive psychology – the study of human strength and optimal functioning. In contrast to the MBI, the UWES measures positive feelings such as vigor, dedication, and absorption in one’s job.^36^ The UWES attempts to measure burnout by measuring the opposite of burnout, with the underlying assumption that engaged workers have a positive, fulfilling, work-related state of mind. Multiple studies have demonstrated that work engagement is significantly inversely related to burnout.^37^ It should be noted that the same group of researchers who developed the MBI also developed the UWES.

The UWES has been studied in very large populations and has been validated in non-U.S. physicians.^38^–^40^ It is also available in multiple languages. However, the normative values are based on a general, Dutch, working population. The UWES is free, easy to use, and can be repeated to monitor progress. This scale could easily be combined with the MBI or the single-item EE and DP scale to measure both physician engagement as well as burnout in order to improve the work environment.

#### Jefferson Scale of Physician Empathy

Empathy is the ability to share and understand the feelings of another. Many neuroscientists believe that empathy is hardwired in human beings and essential to our survival.^41^ Patients want genuine empathy from doctors and doctors want to provide it, but there is a tension in medicine between being able to maintain a healthy detachment from patients while still being able to connect with them.^42^ One of the three key components of burnout is depersonalization, which is not only an inability to feel empathy for others but a loss of connection with oneself. Thus, the ability to measure and monitor empathy in healthcare professionals is important to assessing the degree of depersonalization, one of the major components of burnout.

The Jefferson Scale of Physician Empathy is a validated measure of empathy in healthcare professionals. If decreased empathy, in the form of depersonalization, is a hallmark of burnout, then the routine monitoring of the empathy of healthcare professionals could help to identify loss of compassion that could contribute to burnout.

### Depression and Anxiety Tools

Compared to peers in other fields, medical students and residents experience significantly higher levels of depression. A large study of medical students and residents found that over half screened positive for depression and 8–9% screened positive for suicidal ideation within the prior 12 months.^23^ Another systematic review of 54 studies involving resident physicians (n = 17,560) found that between 20.9% and 43.2% screened positive for depression. Although numerous studies have examined depressive symptoms among medical residents in various specialties, few studies have focused on EM specifically.^43^

Anxiety can be defined as an acute emotional response to stressful conditions (state anxiety), or as a personality characteristic (trait anxiety) that can also be thought of as a predisposition to respond to external threats in fixed ways. Beck’s model of psychopathology suggests that anxiety and depression are separate but related constructs and can be measured independently. In Beck’s definition, anxiety refers to negative feelings that are specific to certain situations, whereas depression involves more absolute and pervasive negative feelings.^44^,^45^ As opposed to the assessments for burnout, the tools for measuring depression and anxiety are validated diagnostic clinical tools widely used for the purpose of identifying DSM-V illnesses as defined by the APA. They are presented with a focus on prevalence and trends within physician populations.

#### Beck Depression Inventory II

The Beck Depression Inventory (BDI-II) is one of the most widely used, self-report measures of depression. The purpose of the BDI-II is to assess the existence and severity of symptoms of depression. Both the total BDI-II score and the single suicidal-ideation items have demonstrated accuracy in predicting suicide attempts and death by suicide.^46^–^50^ The BDI-II has been used in several studies examining depressive symptoms among medical residents in the U.S. and other countries.^51^–^53^

Data from seven studies of resident physicians found the overall prevalence of depression to be 26.6% when using the BDI-II with a cutoff score of ≥ 10. Prevalence of depression was significantly lower among U.S. resident physicians (10.7%) compared to non-U.S. resident physicians (44.6%). The prevalence of depression was higher in more recent studies, and no association was found between prevalence and specialty or post-graduate year (PGY) training level.^54^–^60^ Chronic sleep deprivation was associated with depression.^61^

#### Center for Epidemiologic Studies Depression Scale

The Center for Epidemiologic Studies Depression Scale (CES-D) is commonly used in clinical settings to screen for depression and in research studies with clinical and nonclinical samples.^62^,^63^ Data from seven different studies of resident physicians found the overall prevalence of depression to be 25.6% when using the CES-D and a cutoff score of ≥ 16. Data from two other studies of resident physicians used a higher cutoff of ≥ 19 and found the prevalence to be 33.4%.^64^–^70^ The CES-D is the only measure used in studies examining depressive symptoms of EM residents. A single-site study of 51 EM residents found the prevalence of depression to be 12.1% when using the CES-D and a cutoff of ≥ 15.^71^,^72^ Depression was not associated with gender, rotation type, PGY level, or number of hours worked.^73^

#### Primary Care Evaluations of Mental Disorders: Patient Health Questionnaire and Generalized Anxiety Disorder Instrument

The full Primary Care Evaluation of Mental Disorders (PRIME-MD) and subsequent Patient Health Questionnaire (PHQ) and Generalized Anxiety Disorder Instrument (GAD) were developed as tools for primary care providers to screen for a range of psychiatric disorders, including depression and anxiety. A subsequent shorter version, the Public Health Questionnaire-9 (PHQ-9) is a self-report version of the PRIME-MD depression screen.^74^,^75^ The GAD is the instrument designed to screen for generalized anxiety disorder.^76^ All versions of the PHQ and GAD are considerably shorter and faster to administer than the original PRIME-MD.

The PHQ-9 was used in several studies examining depressive symptoms among residents. Data from four studies of resident physicians found the overall prevalence of depression to be 20.9% when using the PHQ-9. When using a slightly modified version of the PHQ-2 the prevalence of depression among resident physicians was 43.2 %.^77^–^80^ Internal medicine residents who screened positive for depression were more likely to experience burnout^81^ and to report making a medical error.^82^

The PHQ instruments are free and easy to use. With very little training and preparation, clinicians of all types can use these instruments to screen for common psychiatric disorders with relative accuracy. Reliability varies by form, and inter-rater reliability was established on the original PHQ by comparing the use of the instrument by a clinician with assessment of the patient by a mental health professional.^83^ The PHQ instruments do measure depression and anxiety as disorders rather than responses to stress, making it a less favorable instrument for assessing across a physician population.

#### Beck Anxiety Inventory

The Beck Anxiety Inventory (BAI) is a self-report questionnaire that measures severity of anxiety in adults and adolescents. The instrument was specifically designed to “minimize confounding of symptoms of depression.”^45^ The BAI is relatively brief and easy to administer in a short period of time and is most effective as a measurement of somatic symptoms of anxiety.^84^ The instrument does not assess other symptoms of anxiety such as worry or other cognitive aspects and thus may underestimate the presence of anxiety.^85^ The reliability and validity evidence for this instrument has been widely studied; however, the BAI has not been widely studied in medical professional populations.^86^,^87^

#### State-Trait Anxiety Inventory

The State-Trait Anxiety Inventory (STAI) was derived from the Minnesota Multiphasic Personality Inventory (MMPI). The instrument is designed to measure the presence and severity of current symptoms of anxiety and a generalized propensity to be anxious. It is a self-report questionnaire containing two subscales, one for assessing state anxiety (S-Anxiety), questions about how one feels “right now,” and one for assessing trait anxiety (T-Anxiety), questions about how one generally feels.^45^,^93^ The STAI is one of the most widely researched and used measures of general anxiety. The instrument measures both S-anxiety, which is more likely to be prevalent in the emergency department, but it can also measure T-anxiety, illuminating patterns of response to anxiety that may be unhealthy. Because of the overlap of the T-anxiety scale with depression and depressive symptoms, this instrument is limited, having a difficult time achieving respectable levels of discriminant validity. In other words, the T-anxiety scale correlates more with other depression instruments than it does with other measures of anxiety.^94^–^97^

#### Second Victim Experience Support Tool

The Second Victim Experience Support Tool (SVEST) consists of seven subscales that measure psychological distress, physical distress, four types of support, and professional self-efficacy. SVEST also has two outcome measures related to the second victim’s job: intention to leave and absenteeism. The phrase “second victim” refers to healthcare providers who experience an adverse event during the care of their patient, who may be considered the “first victim.”^88^ Medical errors or inadvertent injuries to the patient during care may cause the caregiver to suffer feelings of anxiety, stress, shame, or guilt as a result of adverse clinical event.^89^–^91^ The only major study of the instrument’s psychometric properties was conducted at a pediatric hospital with a very small physician sample.^92^

## LIMITATIONS

There are an overwhelming number of assessment tools available in the literature that can be used to measure the different components of physician well-being. While our literature search was methodical and broad, we acknowledge that we may have missed some key assessment tools. At times, a single author determined the inclusion eligibility of the tools identified in our literature search strategy. However, consensus between at least two reviewers was required for an instrument to be included in this paper.

Assessment tools must be suitable for and validated in the population of interest. A majority of the tools that we found have been used in a physician population but have never been validated in this population. Many of the tools have been designed for and validated in special populations, and their applicability, reliability, and validity in a physician population is not clearly demonstrated in the medical literature. In the absence of independent validation, however, the results of these tools should be interpreted with caution.

Physician well-being is multifactorial, and it is difficult to divide these components purely by topic or sub-category as they have a complex interplay with one another. We have reviewed the tools based on the well-being topics that were most commonly found in the medical literature and that were of highest potential value. There are very few tools that were either designed for use in a physician population or have been validated in physicians. We have highlighted the tools from each topic that are most relevant for use in assessing an EP population.

## CONCLUSION

Given the wide range of associated factors and the psychosocial impact of burnout, it seems unlikely that any one tool will be recognized as comprehensive for evaluating physician well-being. It is hoped that the present review will provide guidance on choosing between currently available instruments, whether assessing mentees or testing interventions in the research setting.

## Supplementary Material





## Figures and Tables

**Figure:**
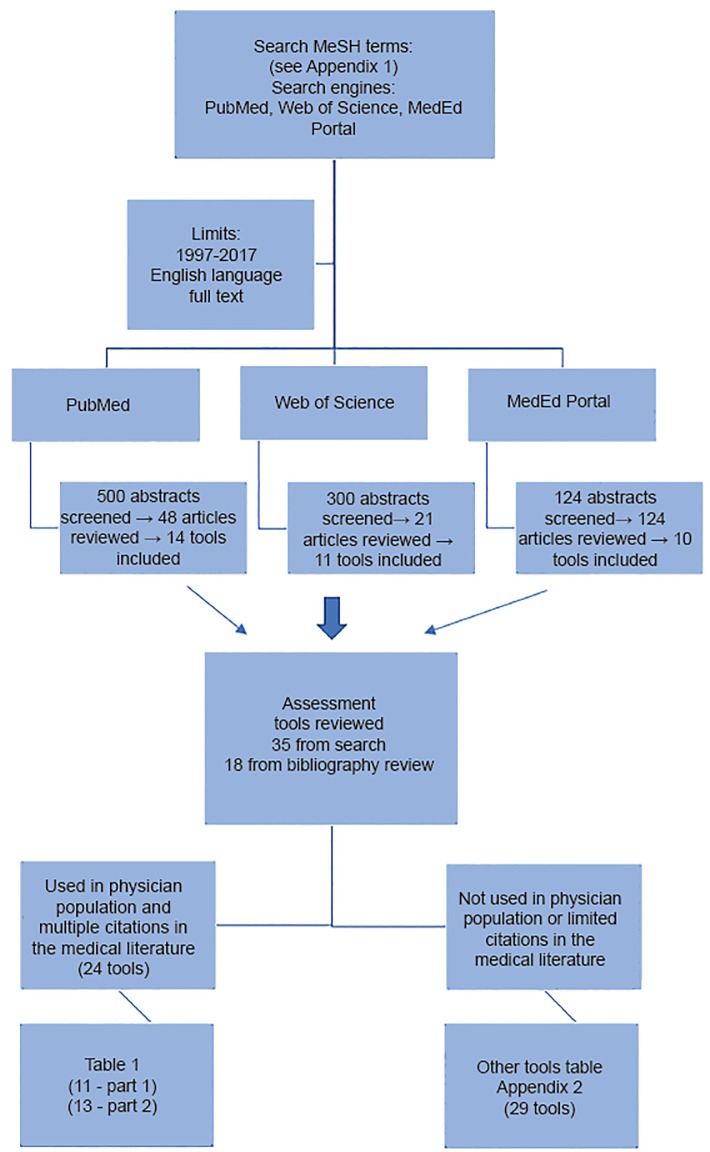
Flow diagram of literature search algorithm and assessment tool selection.

**Table:** Burnout and other negative states assessment tools.

Name of instrument	Brief description	Number of items	Time to complete	Cost	Source	Notes	Pros	Cons
Burnout								
Maslach Burnout Inventory-Health Services Survey (MBI-HSS)	Consists of three subscales: (1) emotional exhaustion (2) depersonalization (3) diminished personal accomplishment.	22 items	10 minutes	$15 per individual report $50 for the manual $250 add-on to calculate and summarize for a group of tests	http://www.mindgarden.com/117-maslach-burnout-inventory Accessed Jan 22, 2019	Wide variability in the interpretation of burnout scores using the MBI has been subject to recent debate. (Rotenstein LS, Torre M, Ramos MA, et al. Prevalence of Burnout Among Physicians: A Systematic Review. *JAMA*. 2018;320(11):1131–50.)	Widely used and well known Developed for human services, such as law enforcement, social work, clergy, and medical professionals	Cost (copyrighted and distributed by a commercial publisher) Variable interpretations of burnout scores
Single item measures of emotional exhaustion (EE) and depersonalization	Consists of only two of the full 22-item MBI questions: “I feel burned out from my work” and “I have become more callous towards people since I took this job.” These questions represent the emotional exhaustion and the depersonalization domains of burnout as described in the MBI, respectively.	Two items	< Two minutes	Free	N/A	Likert Scale responses (1=never, 2=a few times a year, 3=a few times a month, 4=a few times a week 5=once a week, 6=a few times a week, 7=every day). The single scores for the two-item MBI (EE and depersonalization) are multiplied by 9 and 5, respectively.	Ultra-short Free Multiple validation studies	Reliability concerns related to ultra-short assessment tools
Copenhagen Burnout Inventory (CBI)	Consists of 3 sub-dimensions: personal burnout, work-related burnout, and client-related burnout.	19 items	10 minutes	Free	http://www.arbejdsmiljoforskning.dk/upload/cbi-scales.pdf Accessed Jan 22, 2019.	The CBI attempts to distinguish between perceived levels of burnout due to personal factors, work-related factors, and more specifically factors related to work with others.	Free to use Evaluates work-related and patient-related aspects of exhaustion	Single dimension of burnout
Utrecht Work Engagement Scale (UWES)	“Work engagement” is considered to be the antipole of burnout. This scale measures work engagement and arises from the research in positive psychology.	17 items	10 minutes	Free for non-commercial educational and research purposes	https://www.wilmarschaufeli.nl/publications/Schaufeli/Test%20Manuals/Test_manual_UWES_English.pdf Accessed Jan 22, 2019.	Contrary to those who suffer burnout, engaged employees have a sense of energetic and effective connection with their work activities and they see themselves as able to deal well with the demands of their jobs.	Free to use Complements burnout screening	Normative values do not include the United States population
Jefferson Scale of Empathy-Health Professions (JSE-HP)	Measures empathy in healthcare providers and students.	20 items	10 minutes	Approximately $31 per person, scored $5000 for unlimited online use	https://www.jefferson.edu/university/skmc/research/research-medical-education/jefferson-scale-of-empathy.html Order form available: http://www.jefferson.edu/content/dam/university/skmc/research/centerResearch/OrderForm_2016.pdf Accessed Jan 22, 2019.	There are three official versions of the JSE: medical students (S-version), health professions (HP-version), and health professions students (HPS-version). The HP version can be administered to physicians and ALL other health professionals who are involved in patient care, such as nurses, dentists, pharmacists, clinical psychologists, etc.	Well validated Designed for physicians	Cost Indirect measure of wellness
Depression and anxiety								
Beck Depression Inventory-21 item (BDI-II)	Assesses the existence and severity of symptoms of depression; also screens for suicide risk.	21 items	Five minutes	$2.36 per form	http://www.pearsonclinical.com/psychology/products/100000159/beck-depression-inventoryii-bdi-ii.html Accessed Jan 22, 2019.	Assesses symptoms over the preceding two weeks.	Widely used Good psychometric properties	Cost
Center for Epidemiologic Studies Depression Scale (CES-D)	Assesses depression symptoms (utilizing DSM-V criteria) over the last week; also screens for suicide risk.	20 items	2–5 minutes	Free	cesd-r.com/ Accessed Jan 22, 2019.	Developed for use in studies of the epidemiology of depressive symptomatology in the general population.	Free Brief Scale and scoring available online	Reliability concerns
Patient Health Questionnaire (PHQ-2)	Assesses depressive symptoms over the last 2 weeks.	Two items	< Two minutes	Free	http://www.phqscreeners.com/ Accessed Jan 22, 2019.	Derived from the full PHQ which contains the mood, anxiety, alcohol, eating, and somatoform modules.	Ultra-short Free	Reliability concerns related to ultra short assessment tools
Beck Anxiety Inventory (BAI)	Screens for anxiety and describes subjective, somatic, or panic-related symptoms of anxiety.	21 items	5–10 minutes	$2.36 per form	https://www.pearsonclinical.com/psychology/products/100000251/beck-anxiety-inventory-bai.html Accessed Jan 22, 2019.	The BAI has been found to discriminate well between anxious and non-anxious diagnostic groups in a variety of clinical populations.	Validated Good reliability	Costs Not widely studied in health professionals
State-Trait Anxiety Inventory (STAI)	Consists of two subscales, one for assessing State Anxiety (or questions about how one feels “right now”) and one for assessing Trait Anxiety (or questions about how one generally feels).	40 items	10 minutes	$2.50 per form	http://www.mindgarden.com/145-state-trait-anxiety-inventory-for-adults Accessed Jan 22, 2019.	The T-Anxiety scale correlates more with other depression instruments than it does with other measures of anxiety.	Most widely researched and used measure of general anxiety	Cost Overlap with depression and depressive symptoms
Second victim syndrome								
Second Victim Experience Support Tool (SVEST)	Measures psychological distress, physical distress, types of support, and professional self-efficacy. Also measures intention to leave the specialty and absenteeism.	29 items	10–20 minutes	Free for non-commercial educational and research purposes	Burlison JD, et al. The Second Victim Experience and Support Tool. *J Patient Saf*. 2017;13(2):93–102.	Higher scores represent greater likelihood of experiencing second victim characteristics, which include a combination of psychological and physical distress and perceived levels of inadequate support or resources.	Free Novel	Limited studies, need more data on reliability and validity
