# Supplementary material for: Assessment of Physician Well-being, Part One: Burnout and Other Negative States
Source: West J Emerg Med. 2019 Feb 28;20(2):278–90. doi: 10.5811/westjem.2019.1.39665 (PMC6404708; doi:10.5811/westjem.2019.1.39665)
Supplement: Supplementary file 1 [file wjem-20-278-s001.docx]

**Appendix 1.** Search terms for literature search.

**PubMed**

Search Terms:

(((((emotion[TIAB] OR emotion'[TIAB] OR emotion's[TIAB] OR emotion6[TIAB] OR emotiona[TIAB] OR emotionaal[TIAB] OR emotionability[TIAB] OR emotionai[TIAB] OR emotional[TIAB] OR emotional'[TIAB] OR emotional''[TIAB] OR emotionale[TIAB] OR emotionalen[TIAB] OR emotionales[TIAB] OR emotionalinstability[TIAB] OR emotionalised[TIAB] OR emotionalisieren[TIAB] OR emotionalisieren'[TIAB] OR emotionalism[TIAB] OR emotionalist[TIAB] OR emotionalistic[TIAB] OR emotionalitatsinventar[TIAB] OR emotionalities[TIAB] OR emotionality[TIAB] OR emotionality'[TIAB] OR emotionalization[TIAB] OR emotionalize[TIAB] OR emotionalized[TIAB] OR emotionalizes[TIAB] OR emotionalizing[TIAB] OR emotionallly[TIAB] OR emotionally[TIAB] OR emotionally'[TIAB] OR emotionalneglect[TIAB] OR emotionalpersonality[TIAB] OR emotionals[TIAB] OR emotiondiary[TIAB] OR emotione[TIAB] OR emotioned[TIAB] OR emotionen[TIAB] OR emotionful[TIAB] OR emotionful'[TIAB] OR emotiongenic[TIAB] OR emotioning[TIAB] OR emotionis[TIAB] OR emotionl[TIAB] OR emotionladen[TIAB] OR emotionless[TIAB] OR emotionlike[TIAB] OR emotionmemory[TIAB] OR emotionnal[TIAB] OR emotionnalite[TIAB] OR emotionnel[TIAB] OR emotionnelle[TIAB] OR emotionnelles[TIAB] OR emotionnels[TIAB] OR emotiono[TIAB] OR emotionogenic[TIAB] OR emotionol[TIAB] OR emotionology[TIAB] OR emotionregulation[TIAB] OR emotionrelated[TIAB] OR emotions[TIAB] OR emotions'[TIAB] OR emotionsdysregulation[TIAB] OR emotionserkennung[TIAB] OR emotionsregulation[TIAB] OR emotionsregulationsstrategien[TIAB] OR emotionssozialisation[TIAB] OR emotiontional[TIAB] OR emotiontracker[TIAB] OR emotiontropic[TIAB] OR emotionxgroup[TIAB] OR emotionxorder[TIAB]) OR "emotions"[MESH] OR career[TIAB] OR (profession[TIAB] OR profession'[TIAB] OR profession''[TIAB] OR profession's[TIAB] OR profession's'[TIAB] OR professiona[TIAB] OR professionactice[TIAB] OR professionai[TIAB] OR professionais[TIAB] OR professional[TIAB] OR professional'[TIAB] OR professional'nye[TIAB] OR professional's[TIAB] OR professionalas[TIAB] OR professionalcare[TIAB] OR professionalcaretm[TIAB] OR professionalcaretrade[TIAB] OR professionaldocuments[TIAB] OR professionale[TIAB] OR professionalenvironment[TIAB] OR professionales[TIAB] OR professionalgroup[TIAB] OR professionali[TIAB] OR professionalis[TIAB] OR professionalisation[TIAB] OR professionalisation'[TIAB] OR professionalise[TIAB] OR professionalise'[TIAB] OR professionalised[TIAB] OR professionalised'[TIAB] OR professionalises[TIAB] OR professionalising[TIAB] OR professionalising'[TIAB] OR professionalism[TIAB] OR professionalism'[TIAB] OR professionalism''[TIAB] OR professionalism's[TIAB] OR professionalismfemale[TIAB] OR professionalisms[TIAB] OR professionalist[TIAB] OR professionalistic[TIAB] OR professionalists[TIAB] OR professionalitat[TIAB] OR professionalities[TIAB] OR professionalition[TIAB] OR professionalition'[TIAB] OR professionality[TIAB] OR professionalization[TIAB] OR professionalization'[TIAB] OR professionalize[TIAB] OR professionalize'[TIAB] OR professionalized[TIAB] OR professionalized'[TIAB] OR professionalizes[TIAB] OR professionalizing[TIAB] OR professionalizing'[TIAB] OR professionalizm[TIAB] OR professionaljournals[TIAB] OR professionally[TIAB] OR professionally'[TIAB] OR professionallycontacted[TIAB] OR professionalnd[TIAB] OR professionalno[TIAB] OR professionalnursing[TIAB] OR professionalnursing'[TIAB] OR professionals[TIAB] OR professionals'[TIAB] OR professionals'1[TIAB] OR professionals'advice[TIAB] OR professionals'age[TIAB] OR professionals'background[TIAB] OR professionals'dissatisfaction[TIAB] OR professionals'expectations[TIAB] OR professionals'interviews[TIAB] OR professionals'management[TIAB] OR professionals'opinions[TIAB] OR professionals'representationsabout[TIAB] OR professionals'time[TIAB] OR professionals'views[TIAB] OR professionals'well[TIAB] OR professionalsby[TIAB] OR professionalsdagger[TIAB] OR professionalsi[TIAB] OR professionalsim[TIAB] OR professionalsmethods[TIAB] OR professionalsmust[TIAB] OR professionalsto[TIAB] OR professionalstrade[TIAB] OR professionaly[TIAB] OR professionasl[TIAB] OR professionatism[TIAB] OR professionel[TIAB] OR professionell[TIAB] OR professionelle[TIAB] OR professionelle'[TIAB] OR professionellen[TIAB] OR professioneller[TIAB] OR professionelly[TIAB] OR professionels[TIAB] OR professioners[TIAB] OR professionhood[TIAB] OR professioni[TIAB] OR professionials[TIAB] OR professionionals[TIAB] OR professionist[TIAB] OR professionists[TIAB] OR professionitis[TIAB] OR professionl[TIAB] OR professionls[TIAB] OR professionnal[TIAB] OR professionnal's[TIAB] OR professionnalisant[TIAB] OR professionnalisation[TIAB] OR professionnalisme[TIAB] OR professionnals[TIAB] OR professionnals'[TIAB] OR professionnel[TIAB] OR professionnelle[TIAB] OR professionnelles[TIAB] OR professionnelles'[TIAB] OR professionnels[TIAB] OR professionnels'[TIAB] OR professionological[TIAB] OR professionral[TIAB] OR professions[TIAB] OR professions'[TIAB] OR professionships[TIAB] OR professionsl[TIAB] OR professionwide[TIAB]) OR job[TIAB] OR "Professional Practice"[MESH]) AND ("fatigue"[MESH] OR fatigue[TIAB] OR fatigued[TIAB] OR exhausted[TIAB] OR exhaustion[TIAB] OR "Resilience, Psychological"[MESH] OR resilient[TIAB] OR resilience[TIAB] OR resiliency[TIAB] OR "mindfulness"[MeSH Terms] OR (mindful[TIAB] OR mindful'[TIAB] OR mindful2work[TIAB] OR mindfulheart[TIAB] OR mindfullness[TIAB] OR mindfully[TIAB] OR mindfulness[TIAB] OR mindfulness'[TIAB] OR mindfulness's[TIAB] OR mindfulnessand[TIAB] OR mindfulnessbased[TIAB] OR mindfulnessxdepletion[TIAB]) OR "Stress, Psychological"[MESH] OR "frustration"[MESH] OR frustrated[TIAB] OR frustration[TIAB] OR motivation[TIAB] OR occupational stress[TIAB] OR work stress[TIAB] OR "job satisfaction"[MESH] OR job dissatisfaction[TIAB] OR "well being"[TIAB] OR "mental health"[MESH] OR "wellness"[TIAB] OR job distress[TIAB])) OR ("burnout, professional"[MeSH Terms] OR (burnout[TIAB] OR burnout'[TIAB] OR burnout's[TIAB] OR burnout1[TIAB] OR burnoutentstehungsmerkmale[TIAB] OR burnoutmerkmale[TIAB] OR burnouts[TIAB] OR burnouts'[TIAB] OR burnoutsyndrome[TIAB]))) AND ((((("physicians"[MeSH Terms] OR physician[TIAB] OR physicians[TIAB] OR doctor[TIAB] OR doctors[TIAB]) OR ("internship and residency"[MeSH Terms] OR intern[TIAB] OR interns[TIAB] OR (resident[TIAB] OR residents[TIAB] OR residency[TIAB] AND ("physicians"[MeSH Terms] OR physician[TIAB] OR physicians[TIAB])) OR "students, medical"[MeSH Terms] OR (medical student[TIAB] OR medical students[TIAB]))) OR ("Allergists"[MESH] OR (allergist[TIAB] OR allergist's[TIAB] OR allergistis[TIAB] OR allergists[TIAB] OR allergists'[TIAB]) OR "Anesthesiologists"[MESH] OR (anesthesiologist[TIAB] OR anesthesiologist'[TIAB] OR anesthesiologist's[TIAB] OR anesthesiologistes[TIAB] OR anesthesiologistled[TIAB] OR anesthesiologists[TIAB] OR anesthesiologists'[TIAB] OR anesthesiologistsebl[TIAB]) OR "Cardiologists"[MESH] OR (cardiologist[TIAB] OR cardiologist's[TIAB] OR cardiologista[TIAB] OR cardiologistas[TIAB] OR cardiologists[TIAB] OR cardiologists'[TIAB]) OR "Dermatologists"[MESH] OR (dermatologist[TIAB] OR dermatologist's[TIAB] OR dermatologists[TIAB] OR dermatologists'[TIAB] OR dermatologists'management[TIAB] OR dermatologists's[TIAB]) OR "Emergency Medicine"[MESH] OR "Endocrinologists"[MESH] OR (endocrinologist[TIAB] OR endocrinologist's[TIAB] OR endocrinologists[TIAB] OR endocrinologists'[TIAB]) OR "Foreign Medical Graduates"[MESH] OR fmg[TIAB] OR "Gastroenterologists"[MESH] OR (gastroenterologist[TIAB] OR gastroenterologist'[TIAB] OR gastroenterologist's[TIAB] OR gastroenterologists[TIAB] OR gastroenterologists'[TIAB] OR gastroenterologists's[TIAB] OR gastroenterologistsand[TIAB] OR gastroenterologistssurgeons[TIAB]) OR "Gynecology "[MESH] OR (gynecologist[TIAB] OR gynecologist'[TIAB] OR gynecologist's[TIAB] OR gynecologists[TIAB] OR gynecologists'[TIAB] OR gynecologists's[TIAB]) OR Ob-Gyn[TIAB] OR "Hematology"[MESH] OR (hematologist[TIAB] OR hematologist's[TIAB] OR hematologistes[TIAB] OR hematologists[TIAB] OR hematologists'[TIAB]) OR "General Practitioners"[MESH] OR (general practioner[TIAB] OR general practioners[TIAB]) OR GP[TIAB] OR "Geriatricians"[MESH] OR (geriatrician[TIAB] OR geriatrician'[TIAB] OR geriatrician's[TIAB] OR geriatricians[TIAB] OR geriatricians'[TIAB]) OR "Hospitalists"[MESH] OR hospitalist[TIAB] OR hospitalists[TIAB] OR "Nephrologists"[MESH] OR (nephrologist[TIAB] OR nephrologist'[TIAB] OR nephrologist's[TIAB] OR nephrologists[TIAB] OR nephrologists'[TIAB]) OR "Neurologists"[MESH] OR (neurologist[TIAB] OR neurologist's[TIAB] OR neurologists[TIAB] OR neurologists'[TIAB] OR neurologists's[TIAB] OR neurologistsand[TIAB]) OR "Obstetrics"[MESH] OR (obstetric[TIAB] OR obstetric'[TIAB] OR obstetric's[TIAB] OR obstetrica[TIAB] OR obstetrical[TIAB] OR obstetrical'[TIAB] OR obstetricalal[TIAB] OR obstetricalcardiologic[TIAB] OR obstetricale[TIAB] OR obstetricales[TIAB] OR obstetricalgynecological[TIAB] OR obstetrically[TIAB] OR obstetricalneonatal[TIAB] OR obstetricals[TIAB] OR obstetrican[TIAB] OR obstetrican's[TIAB] OR obstetricans[TIAB] OR obstetricanssimilarly[TIAB] OR obstetricare[TIAB] OR obstetricas[TIAB] OR obstetricaux[TIAB] OR obstetricbrachial[TIAB] OR obstetricc[TIAB] OR obstetricepidural[TIAB] OR obstetrices[TIAB] OR obstetricgynecological[TIAB] OR obstetrici[TIAB] OR obstetricia[TIAB] OR obstetricial[TIAB] OR obstetrician[TIAB] OR obstetrician'[TIAB] OR obstetrician's[TIAB] OR obstetriciangynecologist[TIAB] OR obstetriciangynecologists[TIAB] OR obstetricians[TIAB] OR obstetricians'[TIAB] OR obstetriciansand[TIAB] OR obstetricien[TIAB] OR obstetriciens[TIAB] OR obstetricin[TIAB] OR obstetricneonatal[TIAB] OR obstetrico[TIAB] OR obstetricogynecological[TIAB] OR obstetricos[TIAB] OR obstetrics[TIAB] OR obstetrics'[TIAB] OR obstetrics's[TIAB] OR obstetricts[TIAB]) OR "Occupational Health Physicians"[MESH] OR "Ophthalmologists"[MESH] OR (ophthalmologist[TIAB] OR ophthalmologist's[TIAB] OR ophthalmologists[TIAB] OR ophthalmologists'[TIAB] OR ophthalmologists'consultations[TIAB] OR ophthalmologists's[TIAB]) OR "Osteopathic Physicians"[MESH] OR (osteopath[TIAB] OR osteopath's[TIAB] OR osteopathen[TIAB] OR osteopathes[TIAB] OR osteopathia[TIAB] OR osteopathias[TIAB] OR osteopathic[TIAB] OR osteopathic'[TIAB] OR osteopathic's[TIAB] OR osteopathica[TIAB] OR osteopathically[TIAB] OR osteopathie[TIAB] OR osteopathies[TIAB] OR osteopathique[TIAB] OR osteopathist[TIAB] OR osteopathists[TIAB] OR osteopathologic[TIAB] OR osteopathological[TIAB] OR osteopathologies[TIAB] OR osteopathologist[TIAB] OR osteopathology[TIAB] OR osteopathoscopy[TIAB] OR osteopathosis[TIAB] OR osteopaths[TIAB] OR osteopaths'[TIAB] OR osteopathy[TIAB] OR osteopathy'[TIAB] OR osteopathy's[TIAB]) OR "Pathology"[MESH] OR (pathologist[TIAB] OR pathologist'[TIAB] OR pathologist's[TIAB] OR pathologiste[TIAB] OR pathologistes[TIAB] OR pathologistic[TIAB] OR pathologistis[TIAB] OR pathologistological[TIAB] OR pathologists[TIAB] OR pathologists'[TIAB] OR pathologists's[TIAB]) OR "Pediatricians"[MESH] OR (pediatrician[TIAB] OR pediatrician'[TIAB] OR pediatrician'office[TIAB] OR pediatrician's[TIAB] OR pediatriciann[TIAB] OR pediatricians[TIAB] OR pediatricians'[TIAB] OR pediatricians'judgments[TIAB] OR pediatriciansattention[TIAB] OR pediatriciants[TIAB]) OR "Neonatologists"[MESH] OR (neonatologia[TIAB] OR neonatologic[TIAB] OR neonatological[TIAB] OR neonatologically[TIAB] OR neonatologie[TIAB] OR neonatologischen[TIAB] OR neonatologist[TIAB] OR neonatologist'[TIAB] OR neonatologist's[TIAB] OR neonatologistes[TIAB] OR neonatologists[TIAB] OR neonatologists'[TIAB] OR neonatologists's[TIAB] OR neonatologits[TIAB] OR neonatology[TIAB] OR neonatology'[TIAB] OR neonatology's[TIAB] OR neonatologyn[TIAB]) OR "Physiatrists"[MESH] OR (physiatrist[TIAB] OR physiatrist's[TIAB] OR physiatrists[TIAB] OR physiatrists'[TIAB]) OR "Psychiatry"[MESH] OR "psychiatrist*"[TIAB] OR "Physicians, Family"[MESH] OR "Family Practioner*"[TIAB] OR "Physicians, Primary Care"[MESH] OR (internist[TIAB] OR internist'[TIAB] OR internist's[TIAB] OR internista[TIAB] OR internistas[TIAB] OR interniste[TIAB] OR internisten[TIAB] OR internistendagen[TIAB] OR internistenkongress[TIAB] OR internistenwoche[TIAB] OR internisti[TIAB] OR internistic[TIAB] OR internistical[TIAB] OR internistically[TIAB] OR internistinnen[TIAB] OR internistis[TIAB] OR internistische[TIAB] OR internists[TIAB] OR internists'[TIAB]) OR "Physicians, Women"[MESH] OR "Pulmonologists"[MESH] OR (pulmonologist[TIAB] OR pulmonologist's[TIAB] OR pulmonologists[TIAB] OR pulmonologists'[TIAB]))) OR ("Oncologists"[MESH] OR oncologist[TIAB] OR oncologists[TIAB] OR "radiation oncologists"[MESH] OR (("radiology"[MeSH Terms] OR "radiology"[TIAB] OR "radiography"[MeSH Terms] OR "radiography"[TIAB]) AND oncologist[TIAB]) OR "Rheumatologists"[MESH] OR (rhematologist[TIAB] OR rhematologists[TIAB]))) OR ("Surgeons"[MESH] OR surgeon[TIAB] OR surgeons[TIAB] OR "Barber Surgeons"[MESH] OR "Neurosurgeons"[MESH] OR "Orthopedic Surgeons"[MESH] OR "Urologists"[MESH] OR (urologist[TIAB] OR urologist'[TIAB] OR urologist's[TIAB] OR urologistas[TIAB] OR urologists[TIAB] OR urologists'[TIAB])))) AND ((evaluat[TIAB] OR evaluatability[TIAB] OR evaluatable[TIAB] OR evaluataion[TIAB] OR evaluatation[TIAB] OR evaluatble[TIAB] OR evaluatc[TIAB] OR evaluatd[TIAB] OR evaluate[TIAB] OR evaluate'[TIAB] OR evaluatea[TIAB] OR evaluateclinical[TIAB] OR evaluated[TIAB] OR evaluated'[TIAB] OR evaluated4[TIAB] OR evaluatedafter[TIAB] OR evaluatedahpl[TIAB] OR evaluatedand[TIAB] OR evaluatedbased[TIAB] OR evaluatedble[TIAB] OR evaluatedby[TIAB] OR evaluateded[TIAB] OR evaluatedfor[TIAB] OR evaluatedfurther[TIAB] OR evaluatedi[TIAB] OR evaluatedin[TIAB] OR evaluatedmethanolic[TIAB] OR evaluatedpatients[TIAB] OR evaluatedresults[TIAB] OR evaluatedsignificantly[TIAB] OR evaluatedsix[TIAB] OR evaluatedte[TIAB] OR evaluatedthe[TIAB] OR evaluatedthrough[TIAB] OR evaluatedusing[TIAB] OR evaluatedwere[TIAB] OR evaluatedwith[TIAB] OR evaluatedy[TIAB] OR evaluatedzbso[TIAB] OR evaluatee[TIAB] OR evaluatees[TIAB] OR evaluatees'[TIAB] OR evaluategenetic[TIAB] OR evaluatein[TIAB] OR evaluateing[TIAB] OR evaluatematernal[TIAB] OR evaluatenatural[TIAB] OR evaluatepharma's[TIAB] OR evaluatepost[TIAB] OR evaluater[TIAB] OR evaluatereplacement[TIAB] OR evaluaterisk[TIAB] OR evaluaters[TIAB] OR evaluates[TIAB] OR evaluatesthe[TIAB] OR evaluatestheir[TIAB] OR evaluatet[TIAB] OR evaluateted[TIAB] OR evaluatethe[TIAB] OR evaluatetm[TIAB] OR evaluateur[TIAB] OR evaluateurs[TIAB] OR evaluatezseverity[TIAB] OR evaluati[TIAB] OR evaluatie[TIAB] OR evaluatiing[TIAB] OR evaluatiion[TIAB] OR evaluatijon[TIAB] OR evaluatin[TIAB] OR evaluating[TIAB] OR evaluating'[TIAB] OR evaluatinginflammation[TIAB] OR evaluatingoutcomes[TIAB] OR evaluatingprimary[TIAB] OR evaluatingrhizophagus[TIAB] OR evaluatingthe[TIAB] OR evaluatins[TIAB] OR evaluatio[TIAB] OR evaluatioin[TIAB] OR evaluatiom[TIAB] OR evaluatiomicronn[TIAB] OR evaluation[TIAB] OR evaluation'[TIAB] OR evaluation''[TIAB] OR evaluation's[TIAB] OR evaluation1[TIAB] OR evaluational[TIAB] OR evaluationand[TIAB] OR evaluationappropriate[TIAB] OR evaluationary[TIAB] OR evaluationdagger[TIAB] OR evaluatione[TIAB] OR evaluationed[TIAB] OR evaluationelective[TIAB] OR evaluationfollowing[TIAB] OR evaluationfor[TIAB] OR evaluationg[TIAB] OR evaluationicuintensive[TIAB] OR evaluationii[TIAB] OR evaluationiii[TIAB] OR evaluationin[TIAB] OR evaluationism[TIAB] OR evaluationism'[TIAB] OR evaluationl[TIAB] OR evaluationof[TIAB] OR evaluationproblem[TIAB] OR evaluations[TIAB] OR evaluations'[TIAB] OR evaluationsfragebogen[TIAB] OR evaluationsincluded[TIAB] OR evaluationsinstrument[TIAB] OR evaluationsmethoden[TIAB] OR evaluationsrevealed[TIAB] OR evaluationss[TIAB] OR evaluationsstudie[TIAB] OR evaluationsystems[TIAB] OR evaluationt[TIAB] OR evaluationtions[TIAB] OR evaluationtool[TIAB] OR evaluationwas[TIAB] OR evaluatioon[TIAB] OR evaluatior[TIAB] OR evaluativa[TIAB] OR evaluative[TIAB] OR evaluative'[TIAB] OR evaluativejudgments[TIAB] OR evaluatively[TIAB] OR evaluativeness[TIAB] OR evaluatives[TIAB] OR evaluativism[TIAB] OR evaluativism'[TIAB] OR evaluativist[TIAB] OR evaluativistic[TIAB] OR evaluativo[TIAB] OR evaluatlion[TIAB] OR evaluatng[TIAB] OR evaluaton[TIAB] OR evaluator[TIAB] OR evaluator'[TIAB] OR evaluator's[TIAB] OR evaluators[TIAB] OR evaluators'[TIAB] OR evaluatortrade[TIAB] OR evaluatory[TIAB] OR evaluats[TIAB] OR evaluatt[TIAB] OR evaluatuon[TIAB] OR evaluatyed[TIAB]) OR "evaluation studies"[Publication Type] OR "risk assessment"[MESH] OR "surveys and questionnaires"[MeSH Terms] OR (prevent[TI] OR prevent'[TI] OR preventability[TI] OR preventable[TI] OR preventable'[TI] OR preventables[TI] OR prevental[TI] OR preventation[TI] OR preventative[TI] OR preventatives[TI] OR preventcd[TI] OR prevented[TI] OR preventer[TI] OR preventers[TI] OR preventi[TI] OR preventia[TI] OR preventibility[TI] OR preventible[TI] OR preventicare[TI] OR preventicare's[TI] OR preventie[TI] OR preventif[TI] OR preventif'[TI] OR preventifs[TI] OR preventiion[TI] OR preventilation[TI] OR preventimn[TI] OR preventin[TI] OR preventing[TI] OR preventingchronic[TI] OR preventinginjuries[TI] OR preventio[TI] OR prevention[TI] OR prevention'[TI] OR prevention's[TI] OR preventiona[TI] OR preventional[TI] OR preventionally[TI] OR preventionas[TI] OR preventionhealth[TI] OR preventionist[TI] OR preventionist's[TI] OR preventionists[TI] OR preventionists'[TI] OR preventiono[TI] OR preventionof[TI] OR preventions[TI] OR preventitive[TI] OR preventitve[TI] OR preventiv[TI] OR preventiva[TI] OR preventive[TI] OR preventive'[TI] OR preventively[TI] OR preventives[TI] OR preventiveservices[TI] OR preventivo[TI] OR preventodontic[TI] OR preventodontics[TI] OR preventodontist[TI] OR preventologists[TI] OR preventon[TI] OR preventor[TI] OR preventoria[TI] OR preventorial[TI] OR preventorio[TI] OR preventorium[TI] OR preventorium's[TI] OR preventoriums[TI] OR preventors[TI] OR preventral[TI] OR preventricular[TI] OR preventriculus[TI] OR prevents[TI] OR preventt[TI] OR preventure[TI]) OR (("personality inventory"[MESH] OR personality inventory[TIAB] OR "personality scale"[TIAB]) NOT "prevalence"[MESH] OR prevalence[TIAB]) OR "burnout scale"[TIAB] OR "assessment tool*"[TIAB] OR "assessment scale"[TIAB] OR "assessment inventory"[TIAB] OR (interven[TIAB] OR intervenaao[TIAB] OR intervenability[TIAB] OR intervenable[TIAB] OR intervenaction[TIAB] OR intervenal[TIAB] OR intervenant[TIAB] OR intervenants[TIAB] OR intervenational[TIAB] OR intervencaes[TIAB] OR intervencal[TIAB] OR intervencao[TIAB] OR intervence[TIAB] OR intervenci[TIAB] OR intervencio[TIAB] OR intervencion[TIAB] OR intervencional[TIAB] OR intervencionales[TIAB] OR intervencionalis[TIAB] OR intervenciones[TIAB] OR intervencionismo[TIAB] OR intervencionista[TIAB] OR intervencionistas[TIAB] OR intervencios[TIAB] OR intervencoes[TIAB] OR intervendor[TIAB] OR intervene[TIAB] OR intervene'[TIAB] OR intervened[TIAB] OR intervenee[TIAB] OR interveneional[TIAB] OR interveneous[TIAB] OR intervener[TIAB] OR intervener's[TIAB] OR interveners[TIAB] OR interveners'[TIAB] OR intervenes[TIAB] OR intervenetion[TIAB] OR intervenetions[TIAB] OR intervenewith[TIAB] OR intervenidos[TIAB] OR intervenience[TIAB] OR interveniens[TIAB] OR intervenient[TIAB] OR intervenientes[TIAB] OR intervenienting[TIAB] OR interveniently[TIAB] OR intervenients[TIAB] OR intervenig[TIAB] OR intervening[TIAB] OR intervening'[TIAB] OR intervenion[TIAB] OR intervenions[TIAB] OR intervenir[TIAB] OR intervenition[TIAB] OR interveniton[TIAB] OR intervenolin[TIAB] OR intervenor[TIAB] OR intervenor's[TIAB] OR intervenors[TIAB] OR intervenosa[TIAB] OR intervenosum[TIAB] OR intervenous[TIAB] OR intervenously[TIAB] OR intervenricular[TIAB] OR intervension[TIAB] OR intervensional[TIAB] OR intervensions[TIAB] OR intervensitons[TIAB] OR intervent[TIAB] OR interventaion[TIAB] OR interventation[TIAB] OR interventations[TIAB] OR interventative[TIAB] OR intervente[TIAB] OR intervented[TIAB] OR interventi[TIAB] OR intervential[TIAB] OR interventianal[TIAB] OR interventiaonl[TIAB] OR interventicrnq[TIAB] OR interventicular[TIAB] OR interventiculare[TIAB] OR interventie[TIAB] OR interventies[TIAB] OR interventiion[TIAB] OR interventinal[TIAB] OR interventing[TIAB] OR interventinon[TIAB] OR interventins[TIAB] OR interventinuous[TIAB] OR interventio[TIAB] OR interventiods[TIAB] OR interventiomal[TIAB] OR intervention[TIAB] OR intervention'[TIAB] OR intervention''[TIAB] OR intervention'elles[TIAB] OR intervention's[TIAB] OR interventiona[TIAB] OR interventional[TIAB] OR interventional'[TIAB] OR interventionalbronchoscopic[TIAB] OR interventionalism[TIAB] OR interventionalist[TIAB] OR interventionalist's[TIAB] OR interventionalistic[TIAB] OR interventionalists[TIAB] OR interventionalists'[TIAB] OR interventionalists's[TIAB] OR interventionally[TIAB] OR interventionalpatent[TIAB] OR interventionals[TIAB] OR interventionaltherapies[TIAB] OR interventionaltrade[TIAB] OR interventionare[TIAB] OR interventionary[TIAB] OR interventionboth[TIAB] OR interventionby[TIAB] OR interventionclinicaltrials[TIAB] OR interventiondid[TIAB] OR interventioned[TIAB] OR interventionel[TIAB] OR interventionell[TIAB] OR interventionelle[TIAB] OR interventionellen[TIAB] OR interventionem[TIAB] OR interventionen[TIAB] OR interventionentitled[TIAB] OR interventionexternal[TIAB] OR interventionfor[TIAB] OR interventiongroup[TIAB] OR interventionin[TIAB] OR interventioning[TIAB] OR interventionis[TIAB] OR interventionism[TIAB] OR interventionist[TIAB] OR interventionist's[TIAB] OR interventionistic[TIAB] OR interventionists[TIAB] OR interventionists'[TIAB] OR interventionl[TIAB] OR interventionmodels[TIAB] OR interventionnal[TIAB] OR interventionnel[TIAB] OR interventionnelle[TIAB] OR interventionnelles[TIAB] OR interventionniste[TIAB] OR interventionon[TIAB] OR interventionone[TIAB] OR interventionoutcomesplow[TIAB] OR interventionpatients[TIAB] OR interventionplanner[TIAB] OR interventionpregnant[TIAB] OR interventionrelated[TIAB] OR interventionresearch[TIAB] OR interventionrr[TIAB] OR interventions[TIAB] OR interventions'[TIAB] OR interventions4[TIAB] OR interventionsa[TIAB] OR interventionsaimed[TIAB] OR interventionsamong[TIAB] OR interventionsand[TIAB] OR interventionschool[TIAB] OR interventionsdiffering[TIAB] OR interventionseducational[TIAB] OR interventionseffekte[TIAB] OR interventionsfocusing[TIAB] OR interventionsfor[TIAB] OR interventionsforschung[TIAB] OR interventionsforsupratentorial[TIAB] OR interventionsin[TIAB] OR interventionsinclude[TIAB] OR interventionsinpatient[TIAB] OR interventionskonzepte[TIAB] OR interventionson[TIAB] OR interventionsone[TIAB] OR interventionsoriginal[TIAB] OR interventionsoutcomedesignqualityconsistencydirectnessoverall[TIAB] OR interventionsphysical[TIAB] OR interventionsphysician[TIAB] OR interventionsprimary[TIAB] OR interventionsresults[TIAB] OR interventionssectioninterventionconclusion2[TIAB] OR interventionsstudie[TIAB] OR interventionstelephone[TIAB] OR interventionsthat[TIAB] OR interventionsthe[TIAB] OR interventionsthere[TIAB] OR interventionsthese[TIAB] OR interventionsto[TIAB] OR interventionstwo[TIAB] OR interventionsverfahren[TIAB] OR interventionswas[TIAB] OR interventionthe[TIAB] OR interventionthere[TIAB] OR interventiontime[TIAB] OR interventionto[TIAB] OR interventiontreated[TIAB] OR interventionts[TIAB] OR interventionwas[TIAB] OR interventionwe[TIAB] OR interventionwhen[TIAB] OR interventionx[TIAB] OR interventionx2[TIAB] OR interventionxantidepressant[TIAB] OR interventionxtime[TIAB] OR interventios[TIAB] OR interventiosn[TIAB] OR interventioti[TIAB] OR interventiotnal[TIAB] OR interventious[TIAB] OR interventircular[TIAB] OR interventism[TIAB] OR interventistic[TIAB] OR interventistica[TIAB] OR interventivas[TIAB] OR interventive[TIAB] OR interventive'[TIAB] OR intervento[TIAB] OR interventon[TIAB] OR interventonal[TIAB] OR interventons[TIAB] OR interventores[TIAB] OR interventory[TIAB] OR interventral[TIAB] OR interventralis[TIAB] OR interventrcular[TIAB] OR interventrical[TIAB] OR interventricalar[TIAB] OR interventriclar[TIAB] OR interventricle[TIAB] OR interventricualr[TIAB] OR interventriculaire[TIAB] OR interventriculak[TIAB] OR interventricular[TIAB] OR interventriculardelay[TIAB] OR interventriculare[TIAB] OR interventricularia[TIAB] OR interventricularis[TIAB] OR interventricularly[TIAB] OR interventricularseptal[TIAB] OR interventriculo[TIAB] OR interventriculostomy[TIAB] OR interventriculus[TIAB] OR interventrikulares[TIAB] OR interventriuclar[TIAB] OR interventronal[TIAB] OR interventrucular[TIAB] OR intervents[TIAB] OR interventsion[TIAB] OR interventton[TIAB] OR interventuin[TIAB] OR interventuins[TIAB] OR interventus[TIAB] OR intervenus[TIAB]) OR "Cross-Sectional Studies"[MESH] OR "burnout inventory"[TIAB] OR measure[TIAB]) AND (("1997/01/01"[PDAT] : "3000/12/31"[PDAT]) AND English[lang])

PubMed Search #2

Search terms:

(("physicians/psychology"[MAJOR] OR "physician*"[TW] OR "doctor*"[TW] OR "internship and residency"[MeSH Terms] OR "intern"[TW] OR "interns"[TW] OR "resident*"[TW] OR "students, medical"[MeSH Terms] OR (medical student[TW] OR medical students[TW]) OR "Allergists"[MESH] OR (allergist[TIAB] OR allergist's[TIAB] OR allergistis[TIAB] OR allergists[TIAB] OR allergists'[TIAB]) AND "Anesthesiologists"[MESH] OR (anesthesiologist[TIAB] OR anesthesiologist'[TIAB] OR anesthesiologist's[TIAB] OR anesthesiologistes[TIAB] OR anesthesiologistled[TIAB] OR anesthesiologists[TIAB] OR anesthesiologists'[TIAB] OR anesthesiologistsasa[TIAB] OR anesthesiologistsebl[TIAB]) OR "Cardiologists"[MESH] OR (cardiologist[TIAB] OR cardiologist's[TIAB] OR cardiologists[TIAB] OR cardiologists'[TIAB]) OR "Dermatologists"[MESH] OR (dermatologist[TIAB] OR dermatologist's[TIAB] OR dermatologista[TIAB] OR dermatologists[TIAB] OR dermatologists'[TIAB] OR dermatologists'management[TIAB] OR dermatologists's[TIAB]) OR "Endocrinologists"[MESH] OR (endocrinologist[TIAB] OR endocrinologist's[TIAB] OR endocrinologists[TIAB] OR endocrinologists'[TIAB]) OR "Foreign Medical Graduates"[MESH] OR "Gastroenterologists"[MESH] OR (gastroenterologist[TIAB] OR gastroenterologist'[TIAB] OR gastroenterologist's[TIAB] OR gastroenterologists[TIAB] OR gastroenterologists'[TIAB] OR gastroenterologists's[TIAB] OR gastroenterologistsand[TIAB] OR gastroenterologistssurgeons[TIAB]) OR "General Practitioners"[MESH] OR "general practioner*"[TIAB] OR "Geriatricians"[MESH] OR "geriatrician*"[TIAB] OR "Hospitalists"[MESH] OR (hospitalist[TW] OR hospitalist'[TW] OR hospitalist's[TW] OR hospitalistion[TW] OR hospitalistions[TW] OR hospitalists[TW] OR hospitalists'[TW]) OR "Nephrologists"[MESH] OR (nephrologist[TIAB] OR nephrologist'[TIAB] OR nephrologist's[TIAB] OR nephrologists[TIAB] OR nephrologists'[TIAB]) OR "Neurologists"[MESH] OR (neurologist[TIAB] OR neurologist's[TIAB] OR neurologista[TIAB] OR neurologistas[TIAB] OR neurologists[TIAB] OR neurologists'[TIAB] OR neurologists's[TIAB] OR neurologistsand[TIAB]) OR "Occupational Health Physicians"[MESH] OR "Oncologists"[MESH] OR (oncologist[TW] OR oncologist'[TW] OR oncologist's[TW] OR oncologista[TW] OR oncologists[TW] OR oncologists'[TW] OR oncologists'perceptions[TW]) OR "radiation oncologists"[MESH]) AND ("burnout, professional"[MeSH Terms] OR "burnout"[TW] OR "professional burnout"[TW] OR "job burnout"[TW] OR "fatigue"[TW] OR "fatigued"[TW] OR exhausted[TW] OR exhaustion[TW] OR "Resilience, Psychological"[MESH] OR resilient[TW] OR resilience[TW] OR "mindfulness"[MeSH Terms] OR "mindful"[TW] OR "mindfulness"[TW] OR "Stress, Psychological"[MESH] OR "frustration"[MESH] OR "job satisfaction"[MESH] AND ("evaluation studies"[Publication Type] OR "risk assessment"[MESH] OR "surveys and questionnaires"[MeSH Terms] OR (prevent[TW] OR prevent'[TW] OR prevent1[TW] OR prevent2[TW] OR preventa[TW] OR preventa'[TW] OR preventabile[TW] OR preventabilities[TW] OR preventability[TW] OR preventability'[TW] OR preventabl[TW] OR preventable[TW] OR preventable'[TW] OR preventablemost[TW] OR preventables[TW] OR preventably[TW] OR preventaf[TW] OR prevental[TW] OR preventan[TW] OR preventand[TW] OR preventary[TW] OR preventating[TW] OR preventation[TW] OR preventative[TW] OR preventative'[TW] OR preventatively[TW] OR preventatives[TW] OR preventbeta[TW] OR preventblindness[TW] OR preventcancerinfections[TW] OR preventcd[TW] OR preventcoeliacdisease[TW] OR preventd[TW] OR prevente[TW] OR prevented[TW] OR prevented'[TW] OR preventede[TW] OR preventelectrode[TW] OR preventely[TW] OR preventenance[TW] OR preventer[TW] OR preventer'[TW] OR preventers[TW] OR preventers'[TW] OR preventes[TW] OR preventesabeta[TW] OR preventfrequent[TW] OR preventi[TW] OR preventia[TW] OR preventibility[TW] OR preventible[TW] OR preventic[TW] OR preventicare[TW] OR preventicare's[TW] OR preventice[TW] OR preventicon[TW] OR preventics[TW] OR preventicular[TW] OR preventid[TW] OR preventie[TW] OR preventief[TW] OR preventiefonds[TW] OR preventiegids[TW] OR preventiestrategieen[TW] OR preventieve[TW] OR preventif[TW] OR preventif'[TW] OR preventifs[TW] OR preventig[TW] OR preventign[TW] OR preventiion[TW] OR preventilated[TW] OR preventilation[TW] OR preventilator[TW] OR preventilatory[TW] OR preventile[TW] OR preventimaps[TW] OR preventimn[TW] OR preventin[TW] OR preventina[TW] OR preventine[TW] OR preventing[TW] OR preventing'[TW] OR preventing''[TW] OR preventingchronic[TW] OR preventinginjuries[TW] OR preventingmast[TW] OR preventingneuronal[TW] OR preventings[TW] OR preventingthe[TW] OR preventingthem[TW] OR preventingtroilus[TW] OR preventingvarious[TW] OR preventinmaternal[TW] OR preventio[TW] OR preventioin[TW] OR prevention[TW] OR prevention'[TW] OR prevention's[TW] OR prevention3[TW] OR preventiona[TW] OR preventional[TW] OR preventionally[TW] OR preventionamong[TW] OR preventionand[TW] OR preventionas[TW] OR preventioncontrol[TW] OR preventiondod[TW] OR preventioneffects[TW] OR preventiong[TW] OR preventionhealth[TW] OR preventionist[TW] OR preventionist's[TW] OR preventionistic[TW] OR preventionists[TW] OR preventionists'[TW] OR preventionjohn[TW] OR preventionk[TW] OR preventiono[TW] OR preventionof[TW] OR preventionofchronic[TW] OR preventionp[TW] OR preventionpppm[TW] OR preventionrecommended[TW] OR preventions[TW] OR preventions'[TW] OR preventionsuccessful[TW] OR preventionworksar[TW] OR preventitious[TW] OR preventitive[TW] OR preventitively[TW] OR preventitve[TW] OR preventius[TW] OR preventiv[TW] OR preventiva[TW] OR preventivamente[TW] OR preventivas[TW] OR preventivata[TW] OR preventivato[TW] OR preventive[TW] OR preventive'[TW] OR preventivecare[TW] OR preventivecounseling[TW] OR preventiveintervention[TW] OR preventively[TW] OR preventively'[TW] OR preventivement[TW] OR preventivenutrition[TW] OR preventives[TW] OR preventiveservices[TW] OR preventivi[TW] OR preventiving[TW] OR preventivlagen[TW] OR preventivne[TW] OR preventivnega[TW] OR preventivni[TW] OR preventivnih[TW] OR preventivno[TW] OR preventivnom[TW] OR preventivo[TW] OR preventivos[TW] OR preventix[TW] OR prevently[TW] OR preventment[TW] OR preventmmp[TW] OR preventodontic[TW] OR preventodontics[TW] OR preventodontist[TW] OR preventol[TW] OR preventologists[TW] OR preventon[TW] OR preventor[TW] OR preventoria[TW] OR preventorial[TW] OR preventorio[TW] OR preventorium[TW] OR preventorium's[TW] OR preventoriums[TW] OR preventors[TW] OR preventory[TW] OR preventp[TW] OR preventral[TW] OR preventre[TW] OR preventricular[TW] OR preventricularis[TW] OR preventriculography[TW] OR preventriculostomy[TW] OR preventriculus[TW] OR prevents[TW] OR preventspenicillium[TW] OR preventt[TW] OR preventtable[TW] OR preventthe[TW] OR preventting[TW] OR preventure[TW] OR preventwork[TW] OR preventx[TW]) OR "burnout scale"[All Fields] OR "assessment tool"[All Fields] OR "assessment scale"[All Fields] OR "assessment inventory"[All Fields] OR (("personality inventory"[MeSH Terms] OR ("personality"[All Fields] AND "inventory"[All Fields]) OR "personality inventory"[All Fields] OR "inventory"[All Fields] OR "equipment and supplies"[MeSH Terms] OR ("equipment"[All Fields] AND "supplies"[All Fields]) OR "equipment and supplies"[All Fields]) OR ("weights and measures"[MeSH Terms] OR ("weights"[All Fields] AND "measures"[All Fields]) OR "weights and measures"[All Fields] OR "scale"[All Fields])) NOT ("epidemiology"[Subheading] OR "epidemiology"[All Fields] OR "prevalence"[All Fields] OR "prevalence"[MeSH Terms])))) NOT ("patients"[MeSH Terms] OR "patients"[All Fields])

**Web of Science**

Search terms:

| # 1 | **[283,198](http://apps.webofknowledge.com.proxy.library.emory.edu/summary.do?product=WOS&doc=1&qid=1&SID=4AX9ETgPkERSBtkC8Tc&search_mode=AdvancedSearch&update_back2search_link_param=yes)** | TS=(physician$ OR doctor$)  *Indexes=SCI-EXPANDED, SSCI, A&HCI, CPCI-S, CPCI-SSH, BKCI-S, BKCI-SSH, ESCI, CCR-EXPANDED, IC Timespan=1997-2017* |
| --- | --- | --- |

| # 2 | **[3,125](http://apps.webofknowledge.com.proxy.library.emory.edu/summary.do?product=WOS&doc=1&qid=2&SID=4AX9ETgPkERSBtkC8Tc&search_mode=AdvancedSearch&update_back2search_link_param=yes)** | TS=(physician NEAR/2 intern$) OR TS=(physician NEAR/2 residen*) OR TS=(physician NEAR/2 fellow*)  *Indexes=SCI-EXPANDED, SSCI, A&HCI, CPCI-S, CPCI-SSH, BKCI-S, BKCI-SSH, ESCI, CCR-EXPANDED, IC Timespan=1997-2017* |
| --- | --- | --- |

| # 3 | **[27,046](http://apps.webofknowledge.com.proxy.library.emory.edu/summary.do?product=WOS&doc=1&qid=3&SID=4AX9ETgPkERSBtkC8Tc&search_mode=AdvancedSearch&update_back2search_link_param=yes)** | TS=("medical student$")  *Indexes=SCI-EXPANDED, SSCI, A&HCI, CPCI-S, CPCI-SSH, BKCI-S, BKCI-SSH, ESCI, CCR-EXPANDED, IC Timespan=1997-2017* |
| --- | --- | --- |

| # 4 | **[234,762](http://apps.webofknowledge.com.proxy.library.emory.edu/summary.do?product=WOS&doc=1&qid=4&SID=4AX9ETgPkERSBtkC8Tc&search_mode=AdvancedSearch&update_back2search_link_param=yes)** | TS=(Allergist$ OR Anesthesiologist$ OR Cardiologist$ OR Dermatologist$ OR Endocrinologist$ OR "Foreign Medical Graduates" OR fmg OR Gastroenterologist$ OR "General Practitioner$" OR Geriatricians$ OR Hospitalist$ OR Nephrologist$ OR Oncologist$ OR "radiation oncologist$" OR Rheumatologist$ OR Neurosurgeon$ OR “Orthopedic Surgeon$” OR Urologist$ OR Gastroenterologist$ OR “Gynecologist” OR Ob-Gyn OR Hematologist$ OR "General Practitioner$” OR (GP NOT graphene nanoplatelets) OR Geriatrician$ OR Hospitalist$ OR Nephrologist$ OR Obstetrics OR obstetrician$ OR "Occupational Health Physician$" OR Ophthalmologist$ OR “Osteopathic Physician$” OR Osteopath$ OR pathologist$ OR Pediatrician$ OR neonatolog$ OR Physiatrist$ OR psychiatrist$ OR “family physician$” OR “Family Practioner$” OR “Primary Care Physician$” OR internist$ OR (Women NEAR/2 physician$) OR Pulmonologist$ OR Oncologist$ OR “radiation oncologist$" OR Rheumatologist$ OR Urologist$)  *Indexes=SCI-EXPANDED, SSCI, A&HCI, CPCI-S, CPCI-SSH, BKCI-S, BKCI-SSH, ESCI, CCR-EXPANDED, IC Timespan=1997-2017* |
| --- | --- | --- |

| # 5 | **[128,983](http://apps.webofknowledge.com.proxy.library.emory.edu/summary.do?product=WOS&doc=1&qid=5&SID=4AX9ETgPkERSBtkC8Tc&search_mode=AdvancedSearch&update_back2search_link_param=yes)** | TS=((burnout OR (mental$ NEAR/2 fatigue$)) OR resilien* OR mindful* OR ((job OR work) NEAR/2 frustat$) OR (mental NEAR/2 stress) OR (physician NEAR/2 wellness) OR “burnout syndrome” OR “occupation$ burnout” OR (work NEAR/2 satisf*) OR ((physician$ OR doctor$) NEAR/2 distress) OR (work NEAR/2 stress) OR “career satisfaction” OR “job satisfaction” OR (job NEAR/2 dissatisf*) OR (job NEAR/2 distress) OR (burnout NEAR/2 prevent*) OR (burnout NEAR/2 interven*))  *Indexes=SCI-EXPANDED, SSCI, A&HCI, CPCI-S, CPCI-SSH, BKCI-S, BKCI-SSH, ESCI, CCR-EXPANDED, IC Timespan=1997-2017* |
| --- | --- | --- |

| # 6 | **[14,629,343](http://apps.webofknowledge.com.proxy.library.emory.edu/summary.do?product=WOS&doc=1&qid=6&SID=4AX9ETgPkERSBtkC8Tc&search_mode=AdvancedSearch&update_back2search_link_param=yes)** | TS=(“National survey$” OR index OR (evaluat* NEAR/2 tool$) OR “fit index$” OR "fit indic*" OR measure$ OR assess* OR interven$ OR prevent$ OR questionnaire$ OR *inventor* OR evaluat* OR “personality trait$” OR analy* OR “cross sectional stud*”)  *Indexes=SCI-EXPANDED, SSCI, A&HCI, CPCI-S, CPCI-SSH, BKCI-S, BKCI-SSH, ESCI, CCR-EXPANDED, IC Timespan=1997-2017* |
| --- | --- | --- |

| # 7 | **[484,703](http://apps.webofknowledge.com.proxy.library.emory.edu/summary.do?product=WOS&doc=1&qid=7&SID=4AX9ETgPkERSBtkC8Tc&search_mode=AdvancedSearch&update_back2search_link_param=yes)** | #1 OR #2 OR #3 OR #4  *Indexes=SCI-EXPANDED, SSCI, A&HCI, CPCI-S, CPCI-SSH, BKCI-S, BKCI-SSH, ESCI, CCR-EXPANDED, IC Timespan=1997-2017* |
| --- | --- | --- |

| # 8 | **[4,430](http://apps.webofknowledge.com.proxy.library.emory.edu/summary.do?product=WOS&doc=1&qid=8&SID=4AX9ETgPkERSBtkC8Tc&search_mode=AdvancedSearch&update_back2search_link_param=yes)** | #5 AND #6 AND #7  *Indexes=SCI-EXPANDED, SSCI, A&HCI, CPCI-S, CPCI-SSH, BKCI-S, BKCI-SSH, ESCI, CCR-EXPANDED, IC Timespan=1997-2017* |
| --- | --- | --- |

| # 9 | **[4,278](http://apps.webofknowledge.com.proxy.library.emory.edu/summary.do?product=WOS&doc=1&qid=9&SID=4AX9ETgPkERSBtkC8Tc&search_mode=AdvancedSearch&update_back2search_link_param=yes)** | #5 AND #6 AND #7  **Refined by:** **DOCUMENT TYPES:** ( ARTICLE OR REVIEW )  *Indexes=SCI-EXPANDED, SSCI, A&HCI, CPCI-S, CPCI-SSH, BKCI-S, BKCI-SSH, ESCI, CCR-EXPANDED, IC Timespan=1997-2017* |
| --- | --- | --- |

| # 10 | **[3,955](http://apps.webofknowledge.com.proxy.library.emory.edu/summary.do?product=WOS&doc=1&qid=10&SID=4AX9ETgPkERSBtkC8Tc&search_mode=AdvancedSearch&update_back2search_link_param=yes)** | #5 AND #6 AND #7  **Refined by:** **LANGUAGES:** ( ENGLISH )  *Indexes=SCI-EXPANDED, SSCI, A&HCI, CPCI-S, CPCI-SSH, BKCI-S, BKCI-SSH, ESCI, CCR-EXPANDED, IC Timespan=1997-2017* |
| --- | --- | --- |

| # 11 | **[3,955](http://apps.webofknowledge.com.proxy.library.emory.edu/summary.do?product=WOS&doc=1&qid=11&SID=4AX9ETgPkERSBtkC8Tc&search_mode=AdvancedSearch&update_back2search_link_param=yes)** | #5 AND #6 AND #7  **Refined by:** **LANGUAGES:** ( ENGLISH ) AND **DOCUMENT TYPES:** ( ARTICLE OR REVIEW )  *Indexes=SCI-EXPAANDED, SSCI, A&HCI, CPCI-S, CPCI-SSH, BKCI-S, BKCI-SSH, ESCI, CCR-EXPANDED, IC Timespan=1997-2017* |
| --- | --- | --- |

| # 12 | **[3,955](http://apps.webofknowledge.com.proxy.library.emory.edu/summary.do?product=WOS&doc=1&qid=12&SID=4AX9ETgPkERSBtkC8Tc&search_mode=AdvancedSearch&update_back2search_link_param=yes)** | #5 AND #6 AND #7  **Refined by:** **LANGUAGES:** ( ENGLISH ) AND **DOCUMENT TYPES:** ( ARTICLE OR REVIEW )  *Indexes=SCI-EXPANDED, SSCI, A&HCI, CPCI-S, CPCI-SSH, BKCI-S, BKCI-SSH, ESCI, CCR-EXPANDED, IC Timespan=1997-2017* |
| --- | --- | --- |

**MedEd Portal**

Search terms: Physician Burnout and Burnout; Critical Synthesis Package
