# Supplementary material for: Assessment of Physician Well-being, Part One: Burnout and Other Negative States
Source: West J Emerg Med. 2019 Feb 28;20(2):278–90. doi: 10.5811/westjem.2019.1.39665 (PMC6404708; doi:10.5811/westjem.2019.1.39665)
Supplement: Supplementary file 2 [file wjem-20-278-s002.docx]

| **Name of Instrument** | **Category** | **Brief Description** | **Number of items/ time to complete** | **Source** | **Cost** | **Pros** | **Cons** |
| --- | --- | --- | --- | --- | --- | --- | --- |
| **Burnout** | | | | | |  |  |
| Shirom-Melamed Burnout Measure (SMBM) | Burnout | Assesses burnout using three subscales: physical fatigue, emotional exhaustion, and cognitive weariness. | 14 items  3 min | [http://www.shirom.org/arie/index.html#](http://www.shirom.org/arie/index.html)  Accessed Jan 22, 2019 | Free for non-commercial use | Free  Available in multiple languages  Widely used internationally | No healthcare normative sample  Burnout construct was not meant to be used as a clinically validated diagnosis |
| Oldenburg Burnout Inventory (OLBI) | Burnout | Assesses cognitive and physical components of burnout across 2 dimensions: feelings of exhaustion and disengagement from work. | 16 items  3 min | Demerouti E., Mostert K, Bakker, AB. Burnout and work engagement: A thorough investigation of the independency of both constructs. *J Occup Health Psychol*. 2010;15:209-222. | Free for non-commercial research | Balanced positive and negative wording  Measures burnout and work engagement | There are no well accepted cut-off values for physician scores  Not used as frequently as other scales in the literature |
| Mini-Z Burnout Survey* | Burnout | Determines workplace stress and compare to others’ in the same specialty | 10 items  2 min | <https://edhub.ama-assn.org/data/journals/steps-forward/937327/10.1001stepsforward.2017.0010supp3.docx>  Accessed Jan 22, 2019 | Free | Free  Designed to be utilized by physicians | Must send to AMA for results |
| Physician Job Satisfaction Scale* | Job Satisfaction | Measures physician job satisfaction to assess quality of work-life across medical specialties and settings. | 150 items  25 min | Konrad TR, Willams ES, Linzer M, et al. Measuring physician job satisfaction in a changing workplace and a challenging environment. *Med Care* 1999;37:1174-1182. | Free  With permission | Designed to be utilized by physicians  Available in multiple languages | Length  Considered a work in progress by the authors |
| Psychological Empowerment Instrument | Job Satisfaction | Measures psychological empowerment in the context of the workplace. | 12 items  2 min | <http://webuser.bus.umich.edu/spreitze/Pdfs/EmpowerInstrument.pdf>  Accessed Jan 22, 2019 | Free | Free  Brief | Limited utilization in the medical literature |
| **Resilience/Mindfulness** | | | | | |  |  |
| Brief Resilience Scale (BRS) | Resilience | Measures the ability to bounce back or recover from stress using Likert scale responses. | 6 items  1-5 min | calmhsa.org/wp-content/uploads/2016/02/Brief-Resilience-ScaleEnglish.doc  Accessed Jan 22, 2019  Smith BW, Dalen J, Wiggins K, Tooley E, Christopher P, Bernard J. The brief resilience scale: Assessing the ability to bounce back. *Int J Behav Med*. 2008;15(3) 194-200. | Free | Free  Brief | Developed for use in students, cardiac patients, and chronic pain patients |
| Freiburg Mindfulness Inventory (FMI) | Mindfulness | Measures mindfulness as a unidirectional construct via 14 statements rated on how well they describe the respondent. | 14 items  2 min | <http://www.mindfulness-extended.nl/content3/wp-content/uploads/2013/07/Freiburg-Mindfulness-Inventory.pdf>  Accessed Jan 22, 2019  Walach H, Buchheld N, Buttenmuller V, Kleinknecht N, Schmidt S. Measuring mindfulness - the Freiburg Mindfulness Inventory (FMI). *Personality and Individual Differences*. 2006;40(8) 1543-1555. | Copyrighted  Cost for license to use not published | Brief | Cost  Limited utilization in the medical literature |
| Cognitive and Affective Mindfulness Scale - Revised (CAMS-R) | Mindfulness | Uses a multi-dimensional view of mindfulness as a broad construct including 4 components: attention, present-focus, awareness, and acceptance. | 12 items  2 min | <https://ogg.osu.edu/media/documents/MB%20Stream/CAMS-R.pdf>  Accessed Jan 22, 2019  Feldman, G., Hayes, A., Kumar, S. et al. *J Psychopathol Behav Assess*. 2007;29:177. | Free | Free  Brief  Captures a multi-component measurement of mindfulness | Limited utilization in the medical literature |
| Five Facet Mindfulness Questionnaire (FFMQ) | Mindfulness | Measures trait mindfulness using 5 facets. | 39 items  5-10 min | <http://www.ruthbaer.com/academics/FFMQ.pdf>  Baer RA, Smith GT, Hopkins J, Krietemeyer J, Toney L. Using self-report assessment methods to explore facets of mindfulness. *Assessment*. 2006;13:27-45. | Free | Free  Valid and reliable | Limited utilization in the medical literature |
| Interpersonal Reactivity Index | Empathy | Measures dispositional empathy assuming that empathy consists of a set of separate but related constructs. | 28 items  5 min | <https://www.eckerd.edu/psychology/iri/>  Accessed Jan 22, 2019  Davis, MH. Measuring individual differences in empathy: Evidence for a multidimensional approach. *J Pers Soc Psychol*. 1983;44:113-126. | Free | Free  Published means and standard deviations for adult males and females | Limited utilization in the medical literature |
| Trait Emotional Intelligence Questionnaire* (TEIQue) | Emotional Intelligence | Based on trait emotional intelligence theory and provides a comprehensive assessment of the emotional world of the individual. | 153 items  25 min  Short form available with 30 items, 5 min | <http://www.psychometriclab.com/Home/Default/14>  Accessed Jan 22, 2019  Petrides, KV. Psychometric properties of the Trait Emotional Intelligence Questionnaire. In Stough C, Saklofske DH, Parker JD, Advances in the assessment of emotional intelligence. New York, NY: Springer; 2009. | Free for academic research purposes  Access to normative values and reports have a fee | Free  Available in multiple languages | Length  No additional support provided by the London Psychometric Laboratory |
| **Well-being/Quality of Life** | | | | | |  |  |
| Dupuy Psychological General Well-Being Index | Well-Being | Measures self-representations of intrapersonal affective or emotional states reflecting a sense of subjective well-being or distress. | 22 items  5 min | <https://eprovide.mapi-trust.org/instruments/psychological-general-well-being-index#contact_and_conditions_of_use>  Accessed Jan 22, 2019  Dupuy HJ. The Psychological general Well-Being (PGWB) Index. In: Assessment of Quality of Life in clinical trials of cardiovascular therapies. Edited by Wenger NK, Mattson ME, Furberg CD, Elinson J. Le Jacq Publishing 1984; Chap 9:170-183. | Free for unfunded research  Fee for commercial or funded academic research | Free  Available in multiple languages | Limited utilization in the medical literature |
| Arizona Integrative Outcome Scale (AIOS) | Well-Being | Assesses global sense of spiritual, social, mental, emotional and physical well-being over the last 24 hours and past one month using a visual analogue scale. | 1 item  30 sec | <https://www.ncbi.nlm.nih.gov/pmc/articles/PMC343287/bin/1472-6882-4-1-S1.doc>  Accessed Jan 22, 2019  Bell IR, Cunningham V, Capsi O, Meek P, Ferro L. Development and validation of a new global well-being outcomes rating scale for integrative medicine research. *BMC Complement Altern Med*. 2004;15;4:1. | Free | Free  Brief | Designed for use in integrative medicine research  Limited utilization in the medical literature |
| WHO-5 Well-Being Index | Well-Being | Measures subjective quality of life based on positive mood, vitality, and general interest. | 5 items  2 min | <https://www.psykiatri-regionh.dk/who-5/Pages/default.aspx>  Accessed Jan 22, 2019  Topp CW, Ostergaard SD, Sondergaard S, Bech P. The WHO-5 Well-Being Index: A Systematic Review of the Literature. *Psychotherm Psychosom*. 2015;84:167-176. | Free | Brief  Available in multiple languages  Widely used | Limited utilization in the medical literature  No normative data set for US population |
| Brief Resident Wellness Profile* | Well-Being | Measures residents’ sense of professional accomplishment and mood. | 6 items  1 min | <https://www.utcomchatt.org/docs/Medical_Teacher_2006_Nov_3704_32817.pdf>  Accessed Jan 22, 2019  Keim SM, Mays MZ, Williams JM, Serido J, Harris RB. Measuring wellness among resident physicians. *Medical Teacher*, 2006;28:4, 370-374. | Free | Brief  Designed for use in resident physicians | Limited utilization in the medical literature |
| Resident Well Being (RWB) Scale* | Well-being | Measures resident well-being based on temper, anxiety, attitude, mood, burnout, and work and learning productivity. | 6 items  <5 Min | <https://www.mededportal.org/publication/9405>  Accessed Jan 22, 2019 | Free | Free  Brief  Designed for use in resident physicians | Limited utilization in the medical literature |
| Linear Analog Self-Assessment (QOL) | Quality of Life | Measures 4 major subcomponents of quality of life - physical, emotional, spiritual, and intellectual. | 5 items  2 min | <https://www.ncbi.nlm.nih.gov/pmc/articles/PMC2732111/>  Accessed Jan 22, 2019  Locke DE, Decker PA, Sloan JA, Brown  PD, Malec JF et al. Validation of single-item linear analog scale assessment of quality of life in neuro-oncology patients. *J Pain Symptom Manage*, 2007;34(6):628-38. | Free | Free  Brief | Predominantly utilized in oncology and palliative care patients |
| Optum SF-8 Health Survey Medical Outcomes Study Short Form (SF-8) | Quality of Life | Measures general health concepts not specific to any age, disease, or treatment group. | 8 items  2 min | <https://www.optum.com/solutions/life-sciences/answer-research/patient-insights/sf-health-surveys/sf-8-health-survey.html>  Accessed Jan 22, 2019 | Copyrighted  Cost for license to use not published | Brief  Normative sample available for comparison  Ideal for monitoring population health and in large-scale outcome studies | Limited studies in physicians |
| Pittsburgh Sleep Quality Index (PSQI) | Sleep | Assesses sleep quality and disturbances over a 1-month time period. | 9 items  2 min | <http://www.sleep.pitt.edu/research/instruments.html>  Accessed Jan 22, 2019  Buysse,D.J., Reynolds,C.F., Monk,T.H., Berman,S.R., & Kupfer,D.J. (1989). The Pittsburgh Sleep Quality Index (PSQI): A new instrument for psychiatric research and practice. *Psychiatry Research*, 28(2), 193-213. | Free for non-commercial research and educational research | Free  Brief  Available in multiple languages | Limited studies in physicians  Designed for use in psychiatric patients |
| **Mood/Personality** | | | | | |  |  |
| Positive and Negative Affect Schedule (mood) (PANAS) | Mood | Consists of two 10-item scales to measure both positive and negative affect. | 20 items  2 min | <https://www.statisticssolutions.com/positive-and-negative-affect-schedule-panas/>  Accessed Jan 22, 2019 | Free for non-commercial use  Permission is not required for non-profit research purposes | Free  Brief  Frequent utilization in varied patient populations | Limited studies in physicians |
| Revised Life Orientation Test (LOT-R) | Optimism | Assesses individual differences in generalized optimism versus pessimism. | 10 items  5 min | <http://www.psy.miami.edu/faculty/ccarver/sclLOT-R.html>  Accessed Jan 22, 2019  Carver CS, Scheier MF, Segerstrom SC. Optimism. *Clinical Psychology Review.* 2010;30, 879-889*.* | Free | Free  Brief | No “cut-offs” for optimism or pessimism  Limited studies in physicians |
| Self-Efficacy Questionnaire Scale (SEQS) - Generalized Efficacy Scale | Optimism | Assesses optimistic self-beliefs used to cope with a variety of difficult demands in life. | 10 items  2-3 min | http://userpage.fu-berlin.de/~health/selfscal.htm, <http://userpage.fu-berlin.de/~health/faq_gse.pdf>  Accessed Jan 22, 2019  Schwarzer R & Jerusalem M. Generalized Self-Efficacy scale. In Weinman J, Wright S, Johnston M, eds. *Measures in health psychology: A user’s portfolio. Causal and control beliefs*. Windsor, England: NFER-NELSON;1995. | Free  Must provide citation | Free  Brief  Available in multiple languages  International normative data set available | Limited studies in physicians |
| **Depression/Anxiety/Stress** | | | | | |  |  |
| National Depression Screening Day Scale (HANDS) | Depression | Briefly screens for depression in those attending National Depression Screening Day. | 10 items  2-5 min | <https://www.schneckmed.org/MediaLibraries/SchneckMedical/documents/HANDS-Harvard-NDSD-Screening.pdf>  Accessed Jan 22, 2019  Baer L, Jacobs DG, Meszler-Reizes J, Blais M, Fava M et al. Development of a Brief Screening Instrument: The HANDS. *Psychother Psychosom*. 2000;69:35-41. | Free | Free  Brief | Limited utilization in the medical literature |
| Depression, Anxiety and Stress Scale (DASS-21) (Depression subscale) | Depression | Measures the 3 related negative emotional states of depression, anxiety and tension/stress. | 42 items  5 min | <http://www2.psy.unsw.edu.au/dass/>  Accessed Jan 22, 2019 | Free  Manual is $55 | Free  Brief  Published studies in medical students | Limited studies in physicians |
| Hospital Anxiety and Depression Scale (Depression Subscale) | Depression/  Anxiety | Detects states of depression and anxiety in the setting of a hospital medicine outpatient clinic. | 14 items  2-5 min | http://www.svri.org/sites/default/files/attachments/2016-01-13/HADS.pdf  Accessed Jan 22, 2019  Zigmond AS, Snaith RP. The hospital anxiety and depression scale. *Acta Psychiatr Scand*. 1983;67(6):361-70. | Free | Free  Brief | Limited studies in physicians  Designed for use in outpatients |
| Perceived Medical School Stress Instrument (PMSS) | Stress | Identifies the relative importance of medical school pressures in terms of their relationship with anxiety. | 13 items  10 min | Vitaliano PP, Russo J, Carr JE, Heerwagen JH.. Medical School Pressures and Their Relationship to Anxiety. *J Nerv Ment Dis*. 1984;172(12):730-736. | Free | Free  Designed for use in medical students | Limited utilization in the medical literature in a physician population |
| Measure of Current Status (MOCS) Part A | Stress | Part A items measure participants' current self-perceived status on several skills that are targeted by the intervention: the ability to relax at will, recognize stress-inducing situations, restructure maladaptive thoughts, be assertive about needs, and choose appropriate coping responses as needed. | 13 items  5-10 min | Carver, CS. Measure of Current Status, <http://www.psy.miami.edu/faculty/ccarver/sclMOCS.html>  Accessed Jan 22, 2019  Antoni, M. H., Lechner, S. C., Kazi, A., Wimberly, S. R., Sifre, T., Urcuyo, K. R., Phillips, K., Gluck, S., & Carver, C. S. (2006). How stress management improves quality of life after treatment for breast cancer. *Journal of Consulting and Clinical Psychology, 74,* 1143-1152. | Free | Free  Brief | Limited utilization in the medical literature |
| ***Indicates tool validated in a physician population** | | | | | |  |  |
